# The Arms Race Between *Actinobacillus pleuropneumoniae* and Its Genetic Environment: A Comprehensive Analysis of Its Defensome and Mobile Genetic Elements

**DOI:** 10.1111/mmi.15374

**Published:** 2025-05-03

**Authors:** Giarlã Cunha da Silva, Ciro César Rossi

**Affiliations:** ^1^ Departamento de Microbiologia Universidade Federal de Viçosa Viçosa Brazil; ^2^ Departamento de Bioquímica e Biologia Molecular Universidade Federal de Viçosa Viçosa Brazil

**Keywords:** bacterial evolution, CRISPR, defensome, mobilome, pan‐immune system, pleuropneumonia

## Abstract

*Actinobacillus pleuropneumoniae* is the causative agent of pleuropneumonia in swine, a highly contagious and economically significant disease. The genetic variability of *A. pleuropneumoniae* complicates disease control efforts, as it enables rapid adaptation to various stressors, including antimicrobial treatments. To better understand the molecular mechanisms underlying this adaptability, we investigated the role of the bacterial defensome and its relationship with mobile genetic elements (MGEs), such as prophages, plasmids, and integrative conjugative elements (ICEs). Using bioinformatic tools, we identified a diverse and rich defensome in *A. pleuropneumoniae* , with an average of 16 different defense systems per strain. We found that CRISPR‐Cas systems, along with other defense mechanisms, are actively involved in restricting the entry of foreign genetic material, playing a crucial role in bacterial adaptation. Additionally, we characterized several novel prophages and examined their distribution across different strains, revealing their potential contribution to the bacterium's evolutionary success. Our findings underscore the complex interplay between the bacterium's defense systems and MGEs, shedding light on how *A. pleuropneumoniae* maintains genetic diversity while also safeguarding itself against external threats. These insights provide a better understanding of the genetic factors that influence the pathogen's adaptability and highlight potential avenues for more effective disease control strategies.

## Graphical abstract

Using bioinformatic tools, we identified a diverse defensome in *Actinobacillus pleuropneumoniae* , featuring various defense mechanisms. Genomes show co‐occurring systems like CRISPR, Abi, and restriction‐modification (RM) systems, underscoring the complexity and dynamic nature of their defensome cargo.

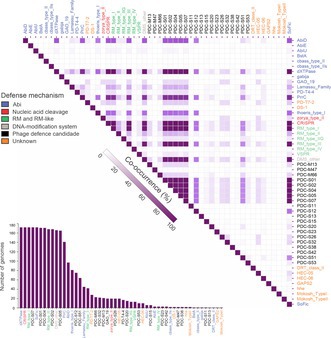

## 1 Introduction

*Actinobacillus pleuropneumoniae* is a significant veterinary pathogen responsible for porcine pleuropneumonia, a severe respiratory disease in pigs that poses high morbidity and can lead to considerable economic losses in animal production. This pathogen affects the swine industry globally, as infected herds experience reduced growth rates, increased mortality, and higher costs associated with veterinary care and preventive measures (Sassu et al. 2018).

The virulence of *A. pleuropneumoniae* is complex and involves multiple factors, such as co‐infection with viral pathogens and specific breeding lineages, but is primarily associated with the production of pore‐forming Apx toxins, which play a key role in its pathogenicity (Pereira et al. 2018; Sassu et al. 2018). Additionally, capsular polysaccharides, outer membrane proteins, nutrient acquisition systems, and extracellular vesicles contribute to its ability to infect and cause disease (Antenucci et al. 2018; Nahar et al. 2021; da Silva et al. 2023). These virulence factors vary among the diverse and growing number of serotypes in which this bacterium is found (Stringer et al. 2021; Seakamela et al. 2024), adding further complexity to understanding and controlling infections caused by *A. pleuropneumoniae* .

Comparative genomics analyses reveal a highly conserved core genome in *A. pleuropneumoniae* , with primary differences arising from the insertion of capsule and lipopolysaccharide clusters, as well as RTX toxin genes, which are often linked to serotype variations (Donà et al. 2022). Genetic variability in this pathogen is largely associated with mobile genetic elements, including integrative and conjugative elements (ICEs), transposons, plasmids, and, notably, bacteriophages. These elements play a significant role in gene acquisition and diversification, contributing to the adaptability, spread of antimicrobial resistance, and pathogenic potential of *A. pleuropneumoniae* across different strains and serotypes (Prado et al. 2020; da Silva et al. 2022).

Bacterial evolution and diversification are profoundly shaped by defense systems collectively known as the bacterial pan‐immune system, or defensome. These mechanisms, which include CRISPR systems, abortive infection (Abi) systems, and restriction‐modification (RM) systems, protect bacteria by detecting and neutralizing foreign genetic material, such as bacteriophage DNA and plasmids, thus enhancing bacterial survival in hostile environments (Bernheim and Sorek 2020). By balancing evolutionary processes, these systems maintain genome stability while allowing selective genetic exchange, influencing gene flow and adaptability within microbial communities (Beavogui et al. 2024). Understanding these defense mechanisms is essential to grasp bacterial evolution, and this study aims to explore the defensome of *A. pleuropneumoniae* , offering insights into the pathogen's resilience and evolutionary strategies.

## 2 Experimental Procedures

### 2.1 Genomes Analyzed

A total of 172 genomes of *A. pleuropneumoniae* representing serovars 1 through 19 were obtained from GenBank and the European Nucleotide Archive (ENA) (last accessed August 2024; Table S1). The serovars of these isolates were confirmed through *in silico* PCR, utilizing the primer sets from Stringer et al. (2021), following the method outlined by Bikandi et al. (2004). To visualize the global distribution of these genomes, we used the ggmap R package (version 4.4.1) to create a heatmap that represents the availability of genomes across different regions based on their origin.

### 2.2 Defense Systems Detection and Analysis

All 172 *A. pleuropneumoniae* genomes were screened for defense systems using the PADLOC (Payne et al. 2022) and DefenseFinder tools (Tesson et al. 2022), and for CRISPR loci with CRISPRCasFinder (Couvin et al. 2018). The distribution of defense systems across isolates was analyzed, generating a presence/absence matrix to assess diversity. A dendrogram was constructed using the PAST software (Hammer et al. 2001) with the Unweighted Pair Group Method with Arithmetic Mean (UPGMA) and the Jaccard index to group isolates based on shared defense system profiles. This approach was chosen to emphasize similarities in defense repertoire composition across strains and serotypes, allowing us to explore potential patterns of horizontal gene transfer and mobility of these systems, which may not always align with phylogenetic relationships. To assess conservation within CRISPR systems, we aligned the Direct Repeat (DR) sequences using Clustal Omega in MEGA X (Tamura et al. 2021), and generated a sequence logo using WebLogo (Crooks et al. 2004).

### 2.3 Co‐Occurrence of Defense System Types

The co‐occurrence of defense systems within the 172 *A. pleuropneumoniae* genomes was analyzed using a binary presence/absence matrix, where the presence (1) or absence (0) of each defense system was recorded for each genome. To assess the frequency of co‐occurrence, a symmetric square matrix was constructed, with both rows and columns representing the total number of defense systems evaluated. The co‐occurrence count was calculated iteratively, with each pair of systems evaluated to determine the number of genomes in which both systems were present simultaneously. The resulting values were stored in the symmetric matrix, and all analyses were conducted using R version 4.4.1.

### 2.4 Identification of Novel Mobile Genetic Elements in *A. pleuropneumoniae*

To identify potential novel mobile genetic elements (MGEs) in *A. pleuropneumoniae* genomes, predictions of integrative conjugative elements (ICEs), plasmids, and phages were performed using ICEfinder (Wang et al. 2024) and Genomad (Camargo et al. 2024). Candidate prophages were compared among themselves and with previously reported prophages in the *Pasteurellaceae* family to eliminate redundancies, using Viridic (Moraru et al. 2020). To confirm the novelty of these candidate prophages, comparative analyses were conducted using the Viral Proteomic Tree server, Viptree (Nishimura et al. 2017). Additionally, the distribution of novel prophages was assessed by aligning these prophages against all genomes included in this study to determine their prevalence and diversity within *A. pleuropneumoniae* .

### 2.5 Analysis of CRISPR'S Spacers Origins

Following the detection of CRISPR systems and putative MGE, all spacer sequences from the CRISPR loci were collected, and redundant sequences were removed using Vsearch (Rognes et al. 2016) with a 95% identity cutoff. The remaining unique spacers were then analyzed by aligning them against a dataset of putative novel MGEs for *A. pleuropneumoniae* (Table S2) and previously documented MGEs from the *Pasteurellaceae* family (Tables S3–S5). Spacer matches were also sought through the IMG/VR and IMG/PR databases from the JGI webserver (Nordberg et al. 2014) to identify viral or plasmid correspondence, respectively. Additionally, Tn central was used to assess potential matches with transposable elements (Ross et al. 2021). For spacers without matches in these databases, a BLAST search against the NCBI database was performed, using “plasmid” and “phage” as filters. For determining spacer origin, we applied a flexible threshold of 80% for both coverage and identity.

### 2.6 Correlation of Co‐Occurrence of MGE and Defense Systems

To examine the relationship between mobile genetic elements (MGEs) and defense systems in *A. pleuropneumoniae* genomes, we analyzed the correlation between the quantity of MGEs detected and the presence of defense systems. First, we calculated the correlation between the number of prophages and the average number of defense systems present in the genomes. Next, we correlated the total count of MGEs with the number of spacers identified for each MGE type (i.e., phage, ICE, and plasmid). Pearson's correlation coefficient was used for these analyses.

### 2.7 Investigation of Defense and Anti‐Defense Systems Located Within MGEs

To assess whether the defense systems could be located within MGEs, all *A. pleuropneumoniae* MGE sequences (including plasmids, prophages, and ICEs from Tables S2–S4) analyzed in this study, both novel and previously reported, were screened for defense systems using the PADLOC (Payne et al. 2022) and DefenseFinder tools (Tesson et al. 2022). Anti‐defense systems in *A. pleuropneumoniae* MGEs were predicted using the dbAPIS database (Yan et al. 2024), while toxin‐antitoxin systems were identified with the TADB 3.0 database (Guan et al. 2024).

### 2.8 Expression of Defense Systems in *A. pleuropneumoniae* in Available RNAseq

To assess the activity of defense systems in *A. pleuropneumoniae* transcriptomes, we analyzed RNAseq data available from the NCBI database. Data for *A. pleuropneumoniae* strains S4074 (serotype 1), JL30 (serotype 3), and SC1810 (serotype 15) were obtained from accession numbers SRR19984988, SRR2074922, and SRR15279005, respectively. Raw reads were processed using Trimmomatic version 0.39 (Bolger et al. 2014) with the following parameters: ILLUMINACLIP.fasta:2:30:10 LEADING:3 TRAILING:3 SLIDINGWINDOW:4:15 MINLEN:40. Reads were then mapped into the *A. pleuropneumoniae* S4074, JL03 and SC1810 reference genomes (GenBank accessions NZ_CP030753.1, NC_010278.1 and NZ_CP071698.1, respectively) using Bowtie2 version 2.4.4 (Langmead and Salzberg 2012), with the “very sensitive” setting. SAM files were converted to BAM format with Samtools version 1.7 (Li et al. 2009), and transcript counts for defense system genes were obtained using Subread version 2.0.2 (Liao et al. 2013). Defense system expression profiles were visualized with Artemis (Carver et al. 2012) or the circlize package in R version 4.4.1. DNA‐modification systems (DMS) and phage defense candidates (PDC) were excluded from this analysis.

## 3 Results

### 3.1 Defense Systems Are Abundant, Widespread, and Co‐Occurring Among *A. pleuropneumoniae* Strains

**FIGURE 1:**
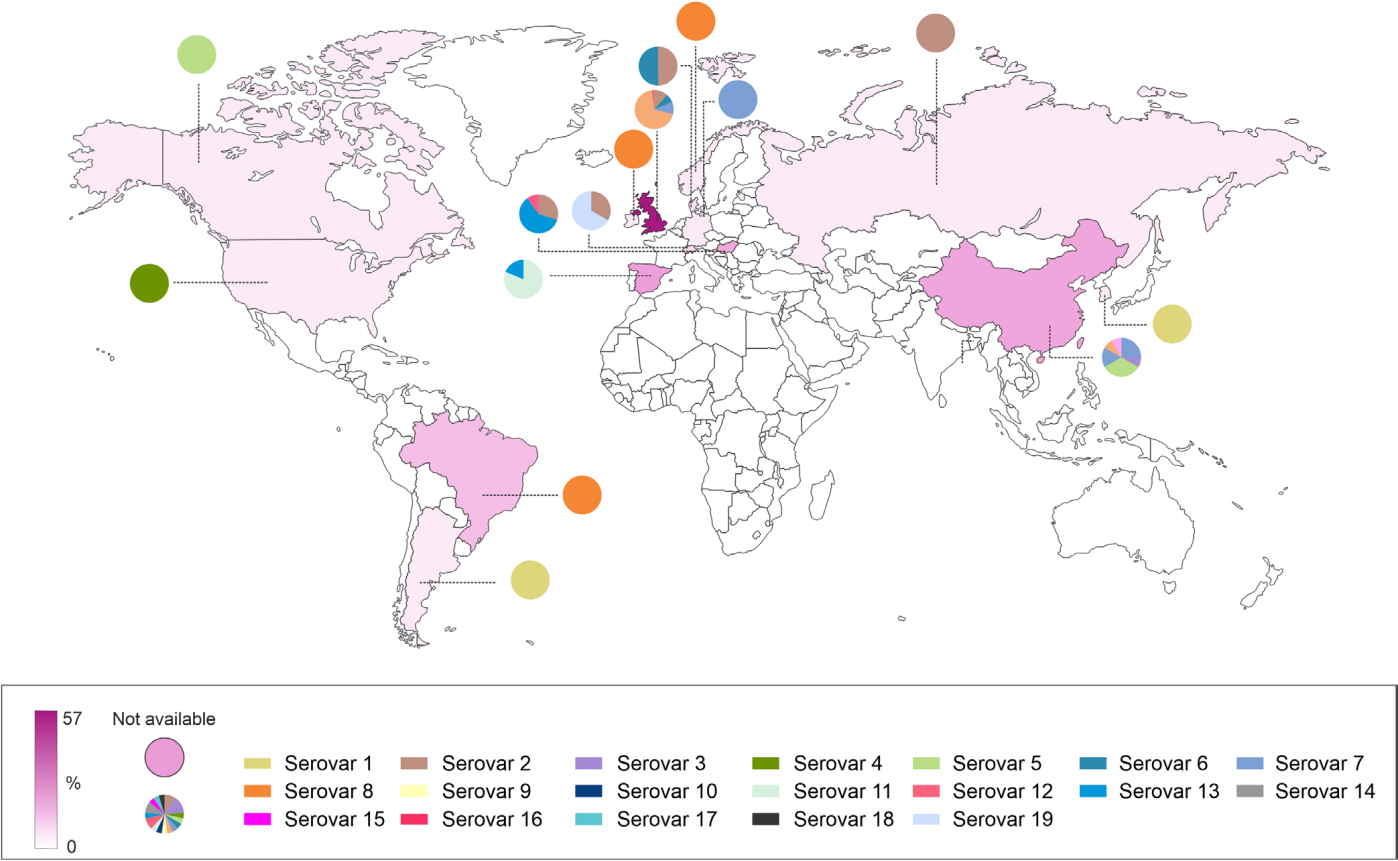
Global distribution of the *Actinobacillus pleuropneumoniae* complete genomes analyzed in this study. Genomes are separated by serotype and strain's isolation area.

This study analyzed 172 complete genomes of *A. pleuropneumoniae* , covering all 19 currently described serotypes. The genomes were sequenced from strains isolated in 15 different countries across Europe, Asia, and the Americas (Figure 1). Serotype 8 was the most prevalent, comprising approximately 44% of the total genomes analyzed. Strains of this serotype were primarily isolated from Western Europe, Brazil, and China.

**FIGURE 2:**
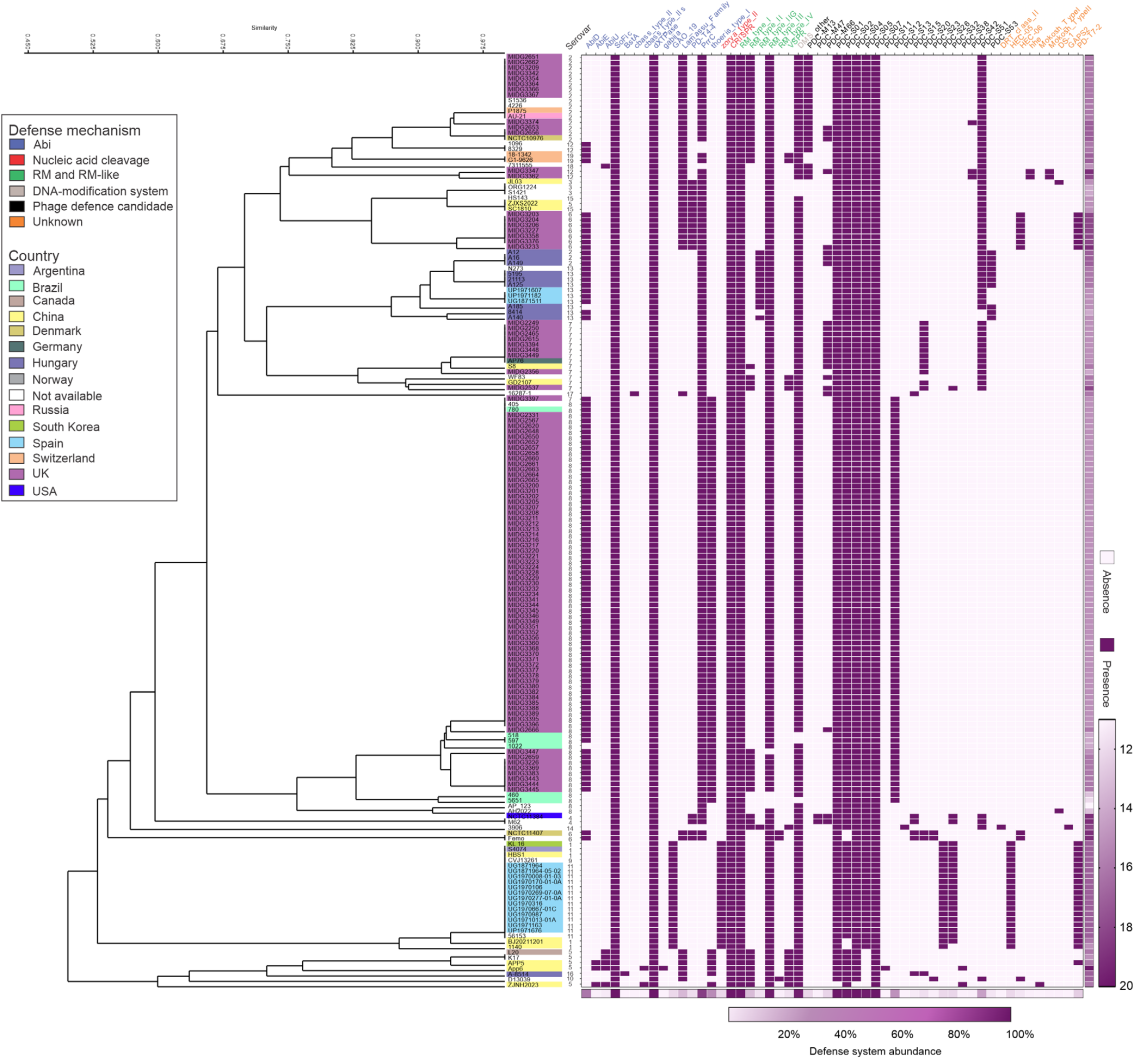
Distribution of defense systems across complete genomes of *Actinobacillus pleuropneumoniae* strains, covering all 19 serotypes and various geographic regions. Strains are organized in a UPGMA dendrogram, built from a presence/absence matrix of defense systems in each isolate. Strains names are color‐coded by region of origin. A heatmap below the dendrogram indicates the abundance of defense systems for each strain, with the total number of systems per strain shown on the right. Defense system names, displayed at the top, are color‐coded by mechanism of action.

A large number of defense systems were identified in the studied genomes, with an average of 16 ± 2 systems per strain (Figure 2 and Table S6). Type I‐F CRISPR systems, the Abi SoFIC system, and dXTPases (defense proteins that halt phage replication by depleting dCTP or dGTP in the bacterial cell) are universal, as they are present in all genomes analyzed. Similarly, 100% of the genomes contain at least one type of restriction‐modification (RM) system. Other Abi mechanisms are also widespread, with the PrrC system found in 80% and AbiD in 58% of the genomes. The distribution of defense systems appears to be consistent among strains of the same serotype, regardless of the region of isolation (Figure 2).

**FIGURE 3:**
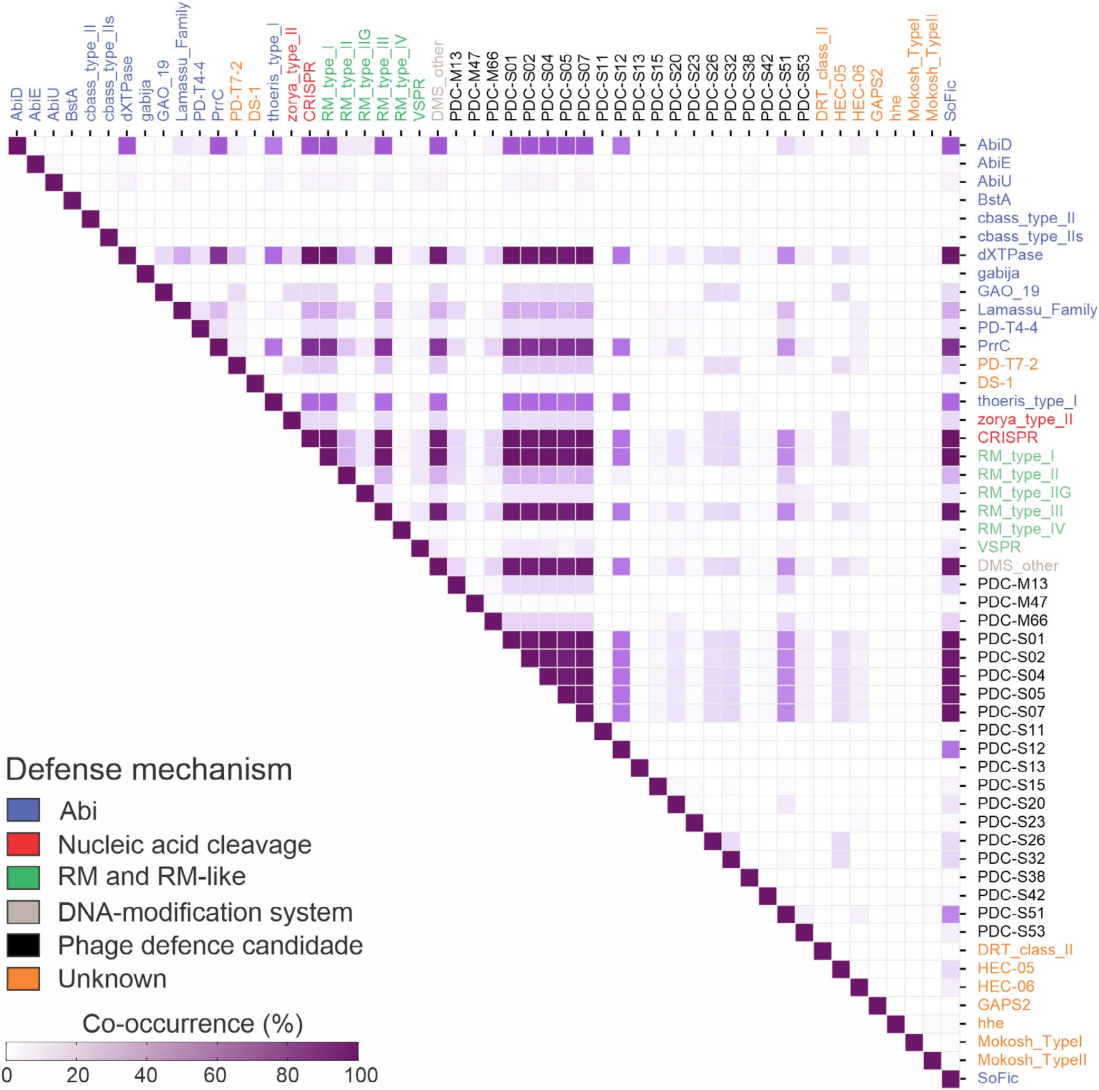
Co‐occurrence of defense systems in *Actinobacillus pleuropneumoniae* . Defense system names are color‐coded based on their mechanism of action.

The analyzed strains also exhibit a combination of co‐occurring systems with different mechanisms. CRISPR systems, along with Abi systems like Thoeris type I, PrrC, and SoFIC, as well as dXTPase and RM types I and III, are frequently found in combination within the same strains (Figure 3). Other, less widespread systems show low or no co‐occurrence with other systems, such as BstA, cBASS, AbiE, AbiU, Gabija, HEC‐06, Hhe, Mokosh, RM type IV, and VSRP.

### 3.2 CRISPR Systems Are Well Conserved and Target Phages and Plasmids

**FIGURE 4:**
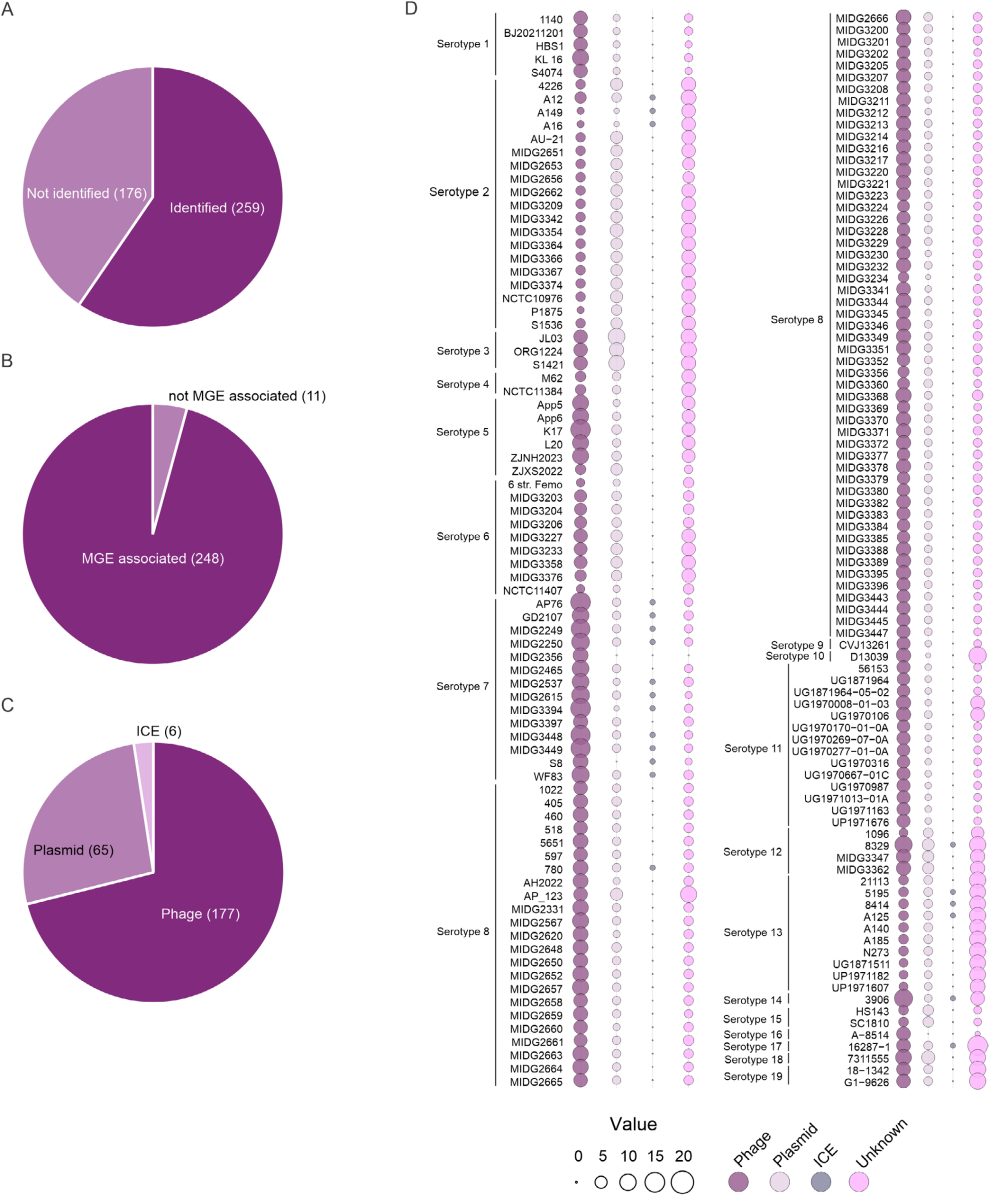
CRISPR spacers in *A. pleuropneumoniae* are predominantly associated with sequences from prophages and plasmids. (A–C) Distribution and likely origin of non‐redundant CRISPR spacers in *A. pleuropneumoniae* . (D) Distribution of spacers' origins in each *A. pleuropneumoniae* isolate, grouped by serotype. The number and correspondence of each circle are detailed at the bottom.

**FIGURE 5:**
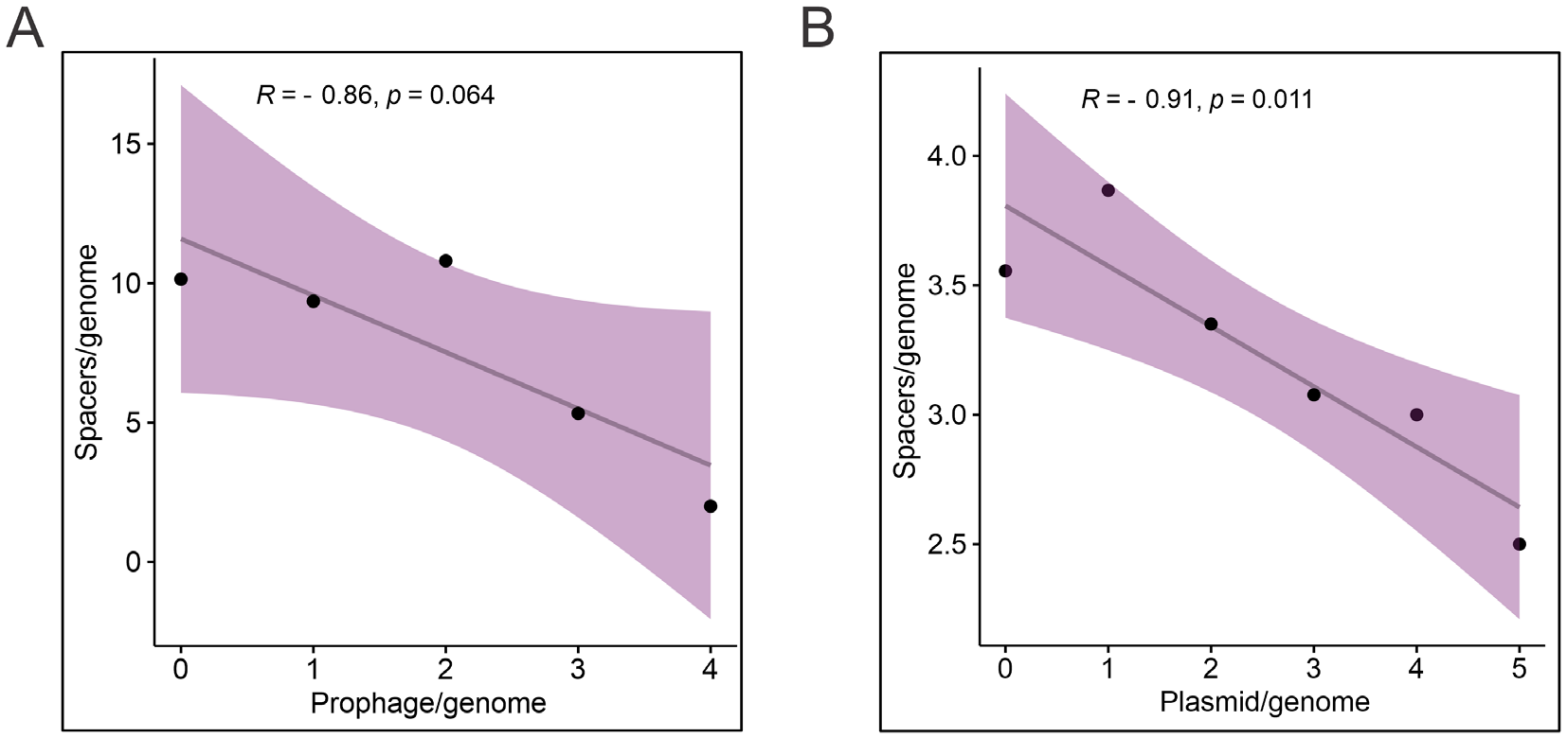
Correlation between mobile genetic elements and spacer presence in *A. pleuropneumoniae* genomes. Pearson's correlation analysis showing the relationship between the average number of spacers matching prophages and the average number of prophages per genome (A) and the average number of spacers matching plasmids and the average number of plasmids per genome (B).

With the exception of strain D13029, from serotype 10 and isolated in China, which has two CRISPR systems (types IF and IIU), all other strains possess only type IF systems. Among systems of the same type, the high sequence conservation (93% identity) suggests a common origin for these systems (Figure S1). In total, 3450 spacers were collected from CRISPR loci, of which 435 were non‐redundant (Table S7). Among these spacers, 258 (59%) showed homology with sequences previously available in databases, allowing identification of their origins (Figure 4A). Most of these, 248 spacers, are associated with known mobile genetic elements (Figure 4B), with 71% of those corresponding to phage sequences, 26% to plasmid sequences, and 3% to integrative and conjugative elements (ICEs) (Figure 4C). Therefore, approximately 97% of spacers with known origins derive from plasmid or bacteriophage sequences (Figure 4D). These results align with the statistically significant negative correlation observed between the average number of spacers present in a strain and the average number of prophages (*p* = 0.064, Figure 5A) and plasmids (*p* = 0.011, Figure 5B) in each genome.

### 3.3 Spacers Appear to Have Preferential Targets and Confer Cross‐Protection Against Various MGEs

**FIGURE 6:**
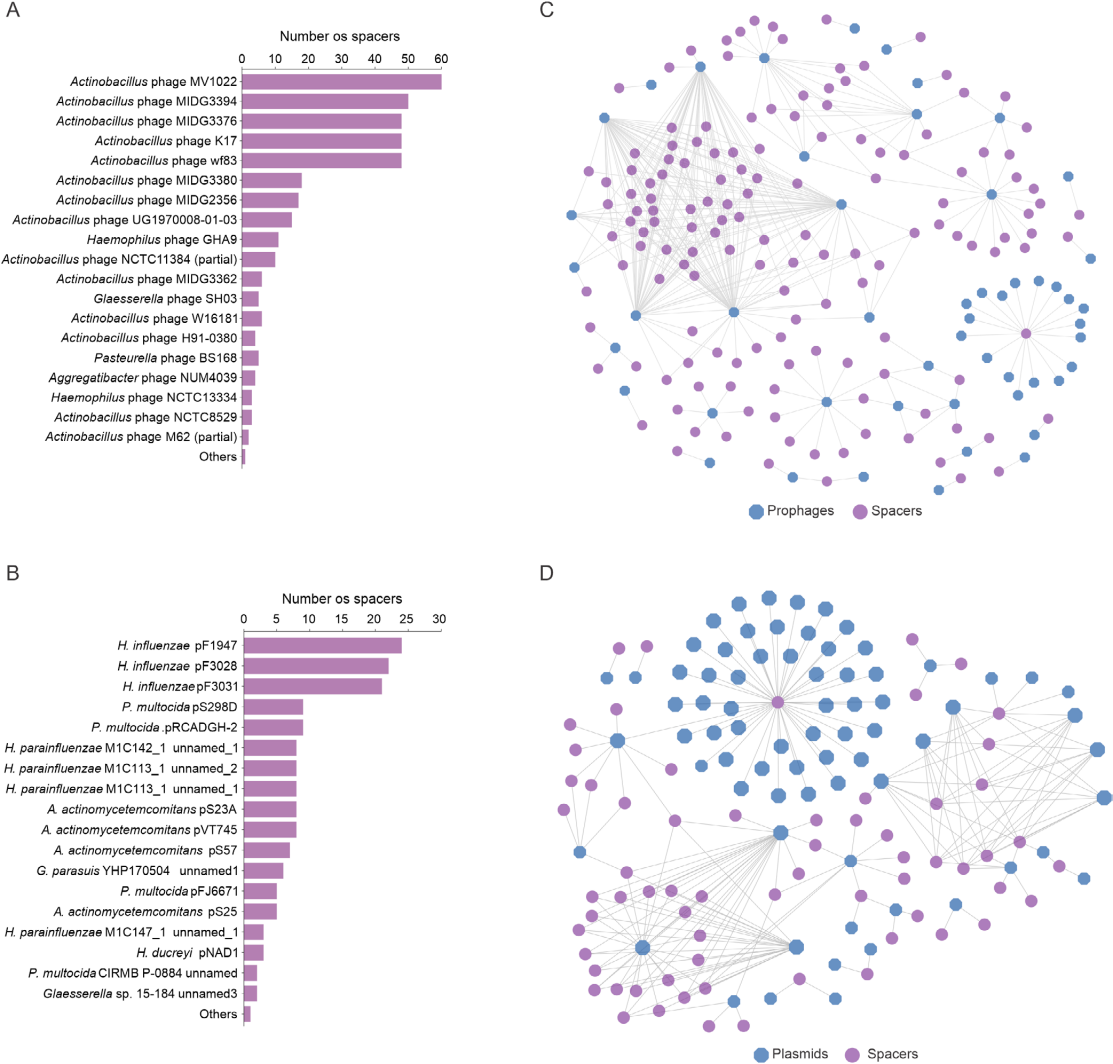
MGEs are frequently recognized by CRISPR, with spacers conferring cross‐protection against MGEs in *A. pleuropneumoniae* . (A) The number of spacers targeting specific prophages. (B) The number of spacers targeting particular plasmids. (C) Network illustrating the correspondence between spacers and prophages and (D) between spacers and plasmids.

A substantial portion of the spacers found targets specific groups of plasmids and bacteriophages available in databases. For instance, among the 435 non‐redundant spacers, approximately 14% target *Actinobacillus* spp. bacteriophages MV1022, MIDG3394, MIDG3376, K17, and WF83 (Figure 6A). Similarly, around 6% (20) of these non‐redundant spacers target fragments of a limited group of plasmids available in databases, including plasmids pF1947, pF3028, and pF3031 (Figure 6B). In some cases, multiple spacers recognize the same elements and provide cross‐protection: several spacers target different bacteriophages and distinct plasmids (Figure 6C,D).

### 3.4 MGEs Also Carry Their Own Defensome

**FIGURE 7:**
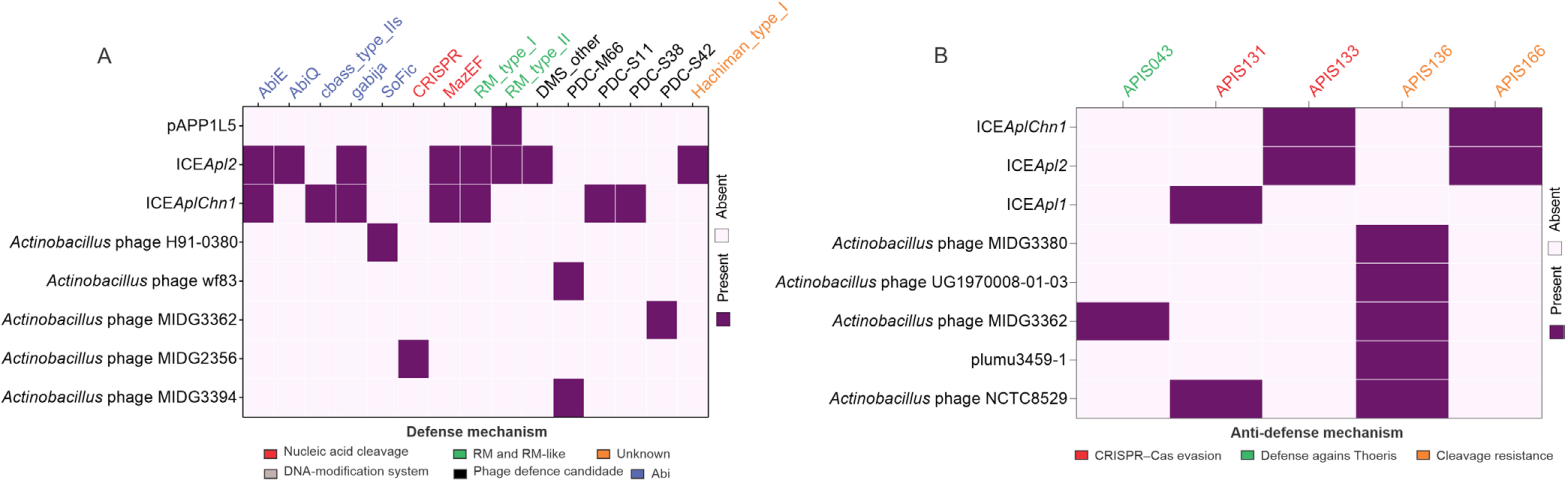
Defense systems (A) and anti‐defense systems (B) present in MGEs from *A. pleuropneumoniae* . Defense systems were identified in both MGEs predicted from the 172 genomes analyzed in this study and in *A. pleuropneumoniae* MGEs previously described in the literature or available in public databases.

To better understand the paradox between the documented role of MGEs in the evolution of *A. pleuropneumoniae* and the abundance of defense systems observed here, we explored whether some of these defense systems could be located within the mobile elements themselves. Indeed, 13 classes of these systems were found within mobile elements, including five prophages, two ICEs, and one plasmid. Among these were Abi systems such as AbiE and CBASS, RM systems, SoFIC, Hachiman, Gabija, DMS, and PDC (Figure 7A and Table S8). Interestingly, the broad‐spectrum anti‐phage system Hachiman (Tuck et al. 2024) was only found in ICE*Apl*2, absent from any of the investigated *A. pleuropneumoniae* genomes. These MGEs also harbor anti‐defense systems, with five different types identified (Figure 7B). The APIS136 system was the most prevalent among phages, while APIS133 and APIS166 were found in ICEs. No anti‐defense systems were detected in plasmids. These systems are associated with evasion of CRISPR‐Cas immunity and protection against cleavage by restriction‐modification (RM) systems. Only one toxin‐antitoxin system, HicA‐HicB, was identified, exclusively in the bacteriophage H91‐0380.

### 3.5 The *A. pleuropneumoniae* Defensome Is Active Under Various Physiological Conditions

**FIGURE 8:**
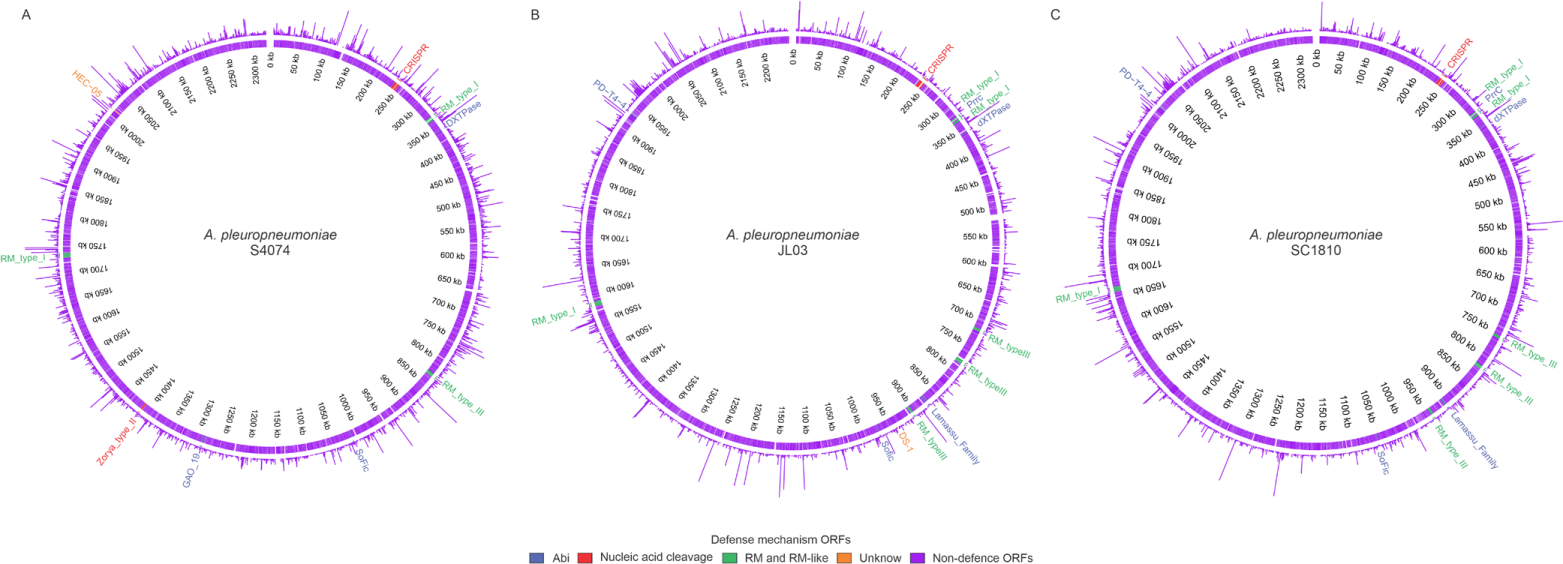
Active Defense Systems in *A. pleuropneumoniae* Strains. Circular representation of transcript levels for defense systems in the genomes of *A. pleuropneumoniae* strains S4074 (A), JL03 (B), and SC1810 (C). The chromosome positions are indicated within the inner circles. Defense system names and corresponding ORFs are color‐coded based on their mechanisms of action.

To assess whether *A. pleuropneumoniae* defense systems are expressed across different conditions, we analyzed publicly available transcriptomes of this bacterium. These include: standard microbial growth conditions, that is, microaerophilic growth without stress induction, for strain JL03 (serotype 03); anaerobic growth in a nitrate‐rich environment for strain S4074 (serotype 1); and exposure to ciprofloxacin and other resistance‐inducing agents for strain SC1810 (serotype 15). Regardless of the condition analyzed, all defense systems, including CRISPR loci with their spacers, were detected as expressed, suggesting that these systems may be constitutively transcribed (Figure 8 and Figure S2).

### 3.6 Novel Prophages Identified in *A. pleuropneumoniae* Genomes

**FIGURE 9:**
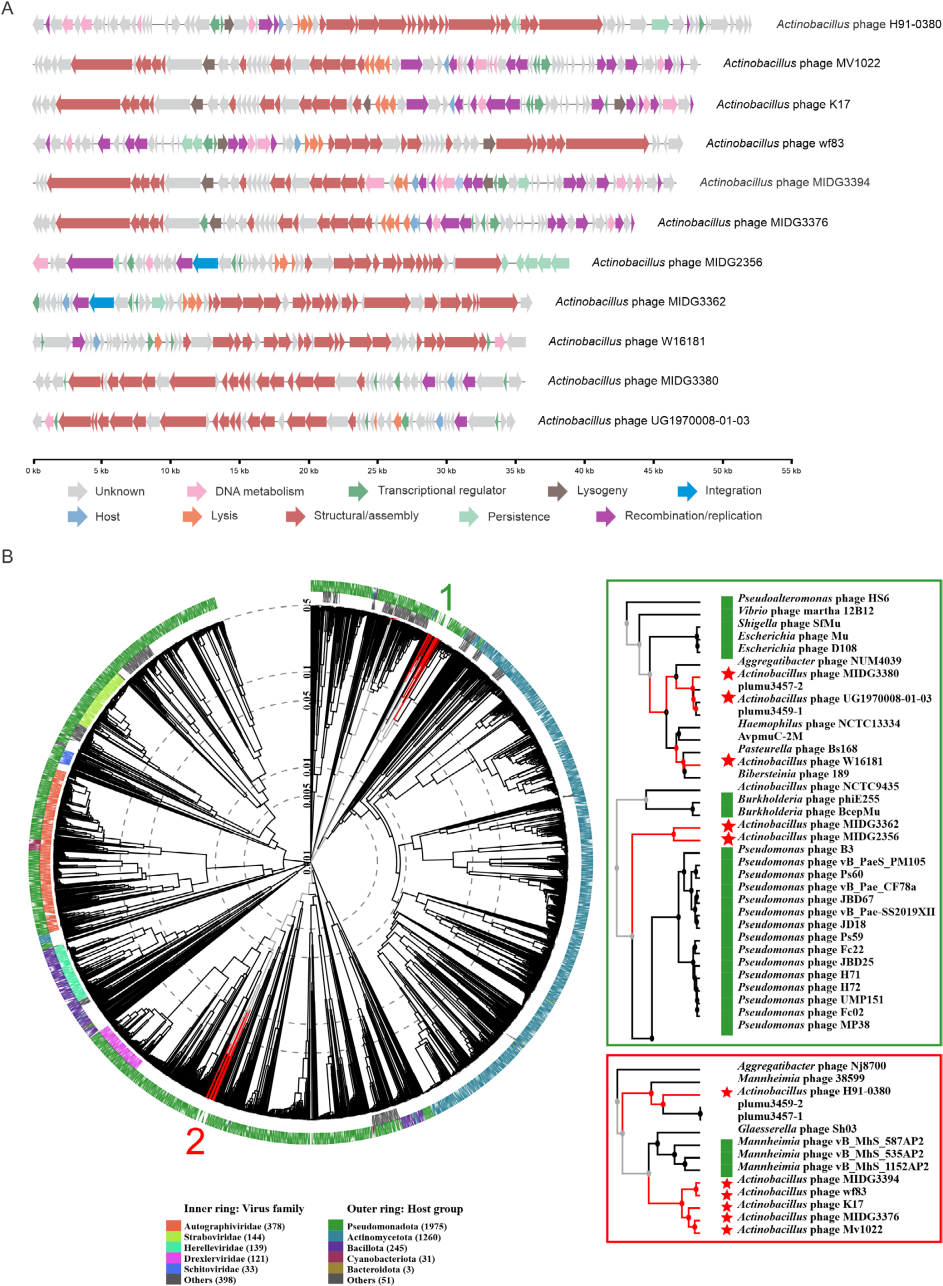
Characterization of novel *Actinobacillus* prophages (A) Schematic representation of novel prophages identified in *A. pleuropneumoniae* genomes. The scale bar indicates prophage size in kilobases, and ORF functions are represented by color‐coded arrows below the figure. (B) Phylogenomic tree of the novel *A. pleuropneumoniae* prophages generated using VipTree. Inner rings indicate viral family classifications, and outer rings represent host groups. Red stars mark the novel prophages identified in this study.

To expand our understanding of novel MGEs in *A. pleuropneumoniae* and enhance spacer identification, we conducted a search for potential new MGEs. Candidate MGEs that matched the CRISPR spacers were further analyzed to confirm novelty. Additionally, uncharacterized prophages identified via IMG/VR were considered for novel phage identification. No new plasmids or ICEs were found, but we identified 14 putative prophage candidates and selected 12 with > 80% completeness for detailed characterization (Table S2). These prophages possess genes encoding proteins involved in assembly and replication, with potential lifestyles classified as either lytic or lysogenic (Figure 9A). Comparative analyses revealed that the 12 candidates are distinct from one another (Figure S3) and differ from previously documented phages in the *Pasteurellaceae* family (Figure 9B and Figure S4). Notably, some of these novel phages are distributed across *A. pleuropneumoniae* genomes analyzed in this study, as demonstrated by the presence of *Actinobacillus* phage MIDG3362, which is present in several strains (Figure S5). No specific pattern of phage integration was observed, except that they are inserted into intergenic regions. Therefore, they do not appear to functionally disrupt adjacent genes.

## 4 Discussion

The global swine industry is a critical sector in animal agriculture, with China, the European Union, the United States, and Brazil playing leading roles and together accounting for more than 75% of global pork production (Szűcs and Vida 2017). In this context, diseases like swine pleuropneumonia, caused by *A. pleuropneumoniae* , present significant economic challenges to the industry (de Conti et al. 2021; Rocha et al. 2022). The high prevalence of serotype 8 is particularly concerning due to its predominance in Chinese, European, and Brazilian swine herds, although it has also been isolated in other regions worldwide (Rossi et al. 2013; Cohen et al. 2021; Seakamela et al. 2024). Serotype 8 is associated with high morbidity and relatively low mortality rates (Pereira et al. 2016), which can hinder early diagnosis and effective treatment, allowing for wider dissemination before detection. In this study, serotype 8 was the most abundant among analyzed genomes, underscoring its relevance in countries where the economic burden of pleuropneumonia is especially pronounced.

Previous comparative genomics studies have begun to shed light on the key characteristics that differentiate *A. pleuropneumoniae* strains across various serotypes, which confer differing degrees of pathogenicity, morbidity, and antimicrobial resistance. An analysis of genomes from 18 of the 19 recognized serotypes revealed a well‐conserved core genome, including a pan‐genome of 4116 genes, of which approximately 42% were present in at least 95% of genomes. The primary differences between serotypes were attributed to mobile genetic elements (MGEs) (Donà et al. 2022). A subsequent study analyzing *A. pleuropneumoniae* genomes, along with other bacteria from the *Pasteurellaceae* family, found that nearly 78% of resistance genes in this group were located within MGEs, including thousands of insertion sequences and bacteriophages (da Silva et al. 2022). This aligns with a detailed study of serotype 8 strains, which showed that more than 50% contained intact prophages, among other sequences, emphasizing the role of these elements in strain diversification (Prado et al. 2020).

The integration of mobile genetic elements (MGEs) into bacterial genomes can provide adaptive advantages, such as antimicrobial resistance and adjustment to diverse environments, but often comes at a fitness cost to the host organism. While, to the best of our knowledge, no direct studies have assessed these trade‐offs in *A. pleuropneumoniae* , evidence from other *Pasteurellaceae* members suggests that similar dynamics may be at play. For instance, San Millan et al. (2010) investigated the fitness cost associated with the pB1000 plasmid, which carries the *bla*_*ROB‐1*_ gene, in *Haemophilus influenzae* . Their findings revealed that, although the plasmid conferred ampicillin resistance, it imposed a competitive disadvantage of approximately 9% per 10 generations in the absence of antibiotic pressure. This underscores the dual nature of MGEs: while they provide beneficial traits, they can also reduce bacterial fitness under non‐selective conditions. Further research with the same plasmid examined the adaptation mechanisms of *Escherichia coli* upon receiving this MGE, demonstrating that insertion sequences (ISs) played a crucial role in mitigating the fitness cost of plasmid carriage (Wedel et al. 2023). This highlights the dynamic interplay between MGEs and host genomes, where genetic elements can evolve to minimize their detrimental impact and enhance persistence within bacterial populations.

On the other hand, the presence of defense systems can also impose a fitness cost on bacteria. At the population level, there seems to be a preference for diversifying the types of defense systems carried by each bacterium rather than maintaining multiple redundant systems (Bernheim and Sorek 2020; Zaayman and Wheatley 2022). Additionally, MGEs may carry their own defense mechanisms, such as CRISPR systems, to ensure their persistence within the bacterial chromosome, thereby avoiding the fitness cost associated with the entry of competing MGEs (Carvalho et al. 2024). Therefore, metabolic costs are linked to both the presence of defense systems and mobile genetic elements, with bacterial populations needing to balance which factor will have the greatest evolutionary impact at a given time. Studies have shown, for example, that Cas protein expression and spacer acquisition can negatively affect bacterial growth (Vale et al. 2015) as well as the minimum inhibitory concentration of certain antimicrobials (Yu et al. 2024).

In light of the compelling indication for the role of MGEs and horizontal gene transfer (HGT) in the genetic variability of *A. pleuropneumoniae* and its adaptability to conditions that lead to pleuropneumonia, this study aimed to contribute further by exploring the role of the bacterial defense systems in this process. These systems, collectively consisting of the bacterial pan‐immune system or defensome, play a critical role in regulating HGT and driving bacterial co‐evolution with MGEs (Bernheim and Sorek 2020; Beavogui et al. 2024). CRISPR‐Cas systems, for instance, provide adaptive immunity by acquiring memory to target and neutralize foreign genetic material. CRISPR‐Cas systems are known to limit plasmid infections besides phage infections (Marraffini and Sontheimer 2008; Hatoum‐Aslan et al. 2014), but specific studies demonstrating this function in *Actinobacillus* or other members of the *Pasteurellaceae* family are currently lacking. RM systems cleave unrecognized DNA sequences, preventing successful MGE integration. Additionally, Abi systems act as a last‐resort defense, causing infected cells to self‐sacrifice to halt MGE propagation (van Houte et al. 2016). However, the impact of these defense systems remains largely unknown in *A. pleuropneumoniae* .

Here we observe that defense systems are abundant and diverse in *A. pleuropneumoniae* , many of which remain poorly characterized, and their exact mechanisms of action are still unknown. Investigating how these systems function at the molecular level represents a promising challenge in bacterial functional genomics. Experimental approaches such as knockout mutants, transcriptomic analyses, and phage/plasmid interference assays could provide valuable insights into the activity and regulation of these defense systems, furthering our understanding of their role in bacterial adaptation and evolution. The observed abundance of defense systems aligns with *A. pleuropneumoniae* 's accessory genome being much smaller than that of other important pathogens in the same family, *Pasteurellaceae*. While the *A. pleuropneumoniae* pan‐genome has around 33% dispensable genes (Donà et al. 2022), *Mannheimia haemolytica* has 68% (Klima et al. 2016), and *Haemophilus influenzae* 62% (Pinto et al. 2019). This suggests that *A. pleuropneumoniae* has a robust defensome that combines diverse defense mechanisms to protect against a wide range of invasive genetic elements. For instance, while CRISPR‐Cas systems are estimated to be present in about 40%–50% of bacterial genomes (Makarova et al. 2015), they are found in 100% of *A. pleuropneumoniae* genomes. In other bacteria highly known for their genome plasticity, such as *Staphylococcus* spp., CRISPR‐Cas abundance can be as low as 3% (Rossi et al. 2017). Additionally, the fact that strains of the same serotype have similar defensomes, regardless of their geographic origin, suggests an evolutionary pressure for the maintenance of these systems.

CRISPR systems in *A. pleuropneumoniae* appear to have been acquired early in its evolutionary history, as indicated by the high conservation of its direct repeats and spacer redundancy. Despite this, the systems seemingly remain active, as shown by the presence of numerous unique spacers. These different spacers must have been acquired during more recent invasions by mobile genetic elements in specific strains. Most identified spacers correspond to bacteriophages, key players in the evolutionary trajectory of *A. pleuropneumoniae* (Prado et al. 2020), although a substantial number are also associated with plasmids. The negative correlation between spacer presence and these two types of MGEs suggests that CRISPR systems do indeed play an important role in limiting the entry of foreign genetic elements in *A. pleuropneumoniae* . The rarity of spacers linked to ICE sequences may be due to the underrepresentation of ICEs in genome annotations, as their identification is often challenging due to their chromosomal integration and structural variability. Moreover, ICEs may encode their own defense systems, which can prevent their recognition and elimination by bacterial immune mechanisms, as illustrated in Figure 7. This self‐protective strategy may reduce the number of CRISPR spacers targeting ICEs, further contributing to their apparent scarcity in our dataset. Still, the large proportion of unidentified spacers points out the yet limited representation of *A. pleuropneumoniae* mobile genetic element sequences in public databases.

We speculate that the observation that multiple spacers recognize the same mobile genetic elements may result from two main factors. First, these CRISPR systems, sharing common origins, lead to overlapping spacers that provide protection against the same elements. Additionally, MGEs themselves may share evolutionary origins, then also contribute to redundant spacer sequences that recognize diverse genetic elements. Despite the well‐known roles of plasmids and bacteriophages in the evolution and adaptation of *A. pleuropneumoniae* to various conditions being acknowledged (da Silva et al. 2017; Michael et al. 2018; Prado et al. 2020; da Silva et al. 2022), there is still a lack of studies exploring their diversity, content, and possible mosaicism in this bacterium. While this is beyond the scope of the present study, we believe that understanding the origins, targets, and redundancy of spacers can begin to shed light on this area.

Since mobile genetic elements can impose a fitness cost on their host cell—though they often provide adaptive advantages—a constant arms race unfolds between the bacterium and its potential invader. Thus, while the bacterium equips itself with a defense arsenal, it is expected that the invader's genome does the same (Koonin et al. 2020). Numerous viral and plasmid defense mechanisms ensure their integration and maintenance, including anti‐CRISPR systems (Pawluk et al. 2018), toxin‐antitoxin systems (Peltier et al. 2020), and mutations allowing them to evade host defenses. Here, we observed that *Actinobacillus* plasmids, prophages, and ICEs also carry their own defensome components, most of which overlap with those found in the bacterial genomes analyzed. Previous studies have shown that many genetic elements integrated into bacterial genomes possess their own defensomes, including CRISPR systems (Rossi et al. 2019; Carvalho et al. 2024), which safeguard their persistence and provide a competitive edge when other foreign elements attempt to enter the cell.

Exemplifying the above observations, we were able to identify a dozen novel, previously uncharacterized prophages in *A. pleuropneumoniae* , many of which are dispersed across several strains of this bacterium. This finding supports the importance of these elements in the evolution of *A. pleuropneumoniae* and suggests that they somehow succeed in evading the bacterial immune system.

In conclusion, our study highlights the complexity and dynamic nature of the *A. pleuropneumoniae* genome, focusing on the ongoing arms race between its defense mechanisms and the role of MGEs in shaping bacterial evolution. We found that the bacterium's rich defensome is widespread and actively involved in restricting the entry of foreign genetic material, contributing to the pathogen's adaptability. Additionally, the identification of novel prophages and their broad distribution across different strains reinforces their significance in the evolutionary success of *A. pleuropneumoniae* . While the extensive repertoire of defense systems provides protection against phages and uncontrolled gene acquisition, it likely comes at a cost. Maintaining multiple defense mechanisms may impose metabolic burdens or limit horizontal gene transfer, potentially restricting genetic flexibility. This trade‐off between protection and adaptability underscores the evolutionary pressures shaping *A. pleuropneumoniae* , warranting further investigation into how these systems are regulated and maintained.

## Author Contributions

**Giarlã Cunha da Silva:** conceptualization, methodology, formal analysis, investigation, writing – original draft, writing – review and editing. **Ciro César Rossi:** conceptualization, formal analysis, writing – original draft, writing – review and editing.

## Conflicts of Interest

The authors declare no conflicts of interest.

## Supporting information


**Figure S1:** Consensus sequence of CRISPR Direct Repeats (DR). The consensus sequence was generated by aligning DRs identified across various CRISPR loci using ClustalW. Each position represents the most frequent nucleotide among the analyzed DRs, with variations indicated beneath the consensus sequence.
**Figure S2:** Expression of CRISPR system in *A. pleuropneumoniae* . RNA‐Seq data from *A. pleuropneumoniae* strains S4074 (top), JL03 (middle), and SC1810 (bottom) display the expression of CRISPR loci, including spacers, across their genomes. Visualization was performed using Artemis.
**Figure S3:** Genome similarity map among the putative novel phages. Heatmap generated by VIRIDIC displaying the percentage identity between pairs of newly identified putative phages. The values within the cells indicate genomic similarity, illustrating the proximity levels among the analyzed phage sequences.
**Figure S4:** Intergenomic similarity map of putative novel phages and previously described phages from *Pasteurellaceae*. Heatmap generated by VIRIDIC displaying the percentage identity between pairs of newly identified putative phages (highlighted in red) and previously described phages. The values within the cells indicate genomic similarity, providing a comparative view of the proximity levels between the novel phages and known phages.
**Figure S5:** Distribution of novel phages across *A. pleuropneumoniae* genomes. Bar graph illustrating the presence of putative novel phages identified in this study across up to 23 different *A. pleuropneumoniae* genomes. This widespread occurrence underscores the potential significance of these phages in the genomic landscape of this species.

**Table S1:** Informations regarding the 172 publicly available *A. pleuropneumoniae* genomes obtained from NCBI and ENA databases.
**Table S2:** Informations of the novel prophages described in this study.
**Table S3:** Informations of reported prophages from *Pasteurellaceae* used in this study.
**Table S4:** Informations of reported ICEs from *Pasteurellaceae* used in this study.
**Table S5:** *Pasteurellacae* plasmids used in this study.
**Table S6:** Defence systems prediction of *A. pleuropneumoniae* genomes using PADLOC and Defensefinder.
**Table S7:** Spacers correspondence of *A. pleuropneumoniae*.
**Table S8:** Defence systems prediction of *A. pleuropneumoniae* MGEs genomes using PADLOC and defensefinder.

## Data Availability

All data are included in the manuscript.
