# Supplementary material for: The Arms Race Between Actinobacillus pleuropneumoniae and Its Genetic Environment: A Comprehensive Analysis of Its Defensome and Mobile Genetic Elements
Source: Mol Microbiol. 2025 May 3;124(1):40–53. doi: 10.1111/mmi.15374 (PMC12242098; doi:10.1111/mmi.15374)
Supplement: Supplementary file 1 — Figure S1: Consensus sequence of CRISPR Direct Repeats (DR). The consensus sequence was generated by aligning DRs identified across various CRISPR loci using ClustalW. Each position represents the most frequent nucleotide among the analyzed DRs, with variations indicated beneath the consensus sequence. Figure S2: Expression of CRISPR system in A. pleuropneumoniae . RNA‐Seq data from A. pleuropneumoniae strains S4074 (top), JL03 (middle), and SC1810 (bottom) display the expression of CRISPR loci, including spacers, across their genomes. Visualization was performed using Artemis. Figure S3: Genome similarity map among the putative novel phages. Heatmap generated by VIRIDIC displaying the percentage identity between pairs of newly identified putative phages. The values within the cells indicate genomic similarity, illustrating the proximity levels among the analyzed phage sequences. Figure S4: Intergenomic similarity map of putative novel phages and previously described phages from Pasteurellaceae. Heatmap generated by VIRIDIC displaying the percentage identity between pairs of newly identified putative phages (highlighted in red) and previously described phages. The values within the cells indicate genomic similarity, providing a comparative view of the proximity levels between the novel phages and known phages. Figure S5: Distribution of novel phages across A. pleuropneumoniae genomes. Bar graph illustrating the presence of putative novel phages identified in this study across up to 23 different A. pleuropneumoniae genomes. This widespread occurrence underscores the potential significance of these phages in the genomic landscape of this species. [file MMI-124-40-s002.docx]

**Supplementary figures**


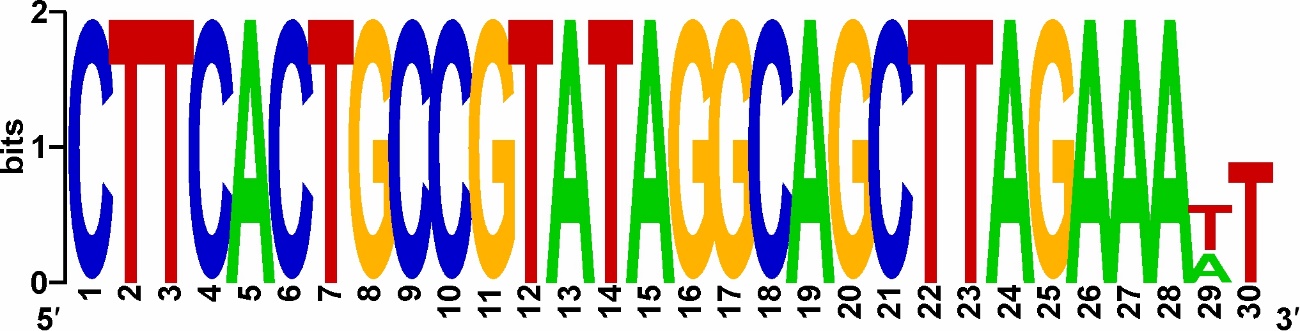


**Supplementary figure S1. Consensus sequence of CRISPR Direct Repeats (DR).** The consensus sequence was generated by aligning DRs identified across various CRISPR loci using ClustalW. Each position represents the most frequent nucleotide among the analyzed DRs, with variations indicated beneath the consensus sequence.


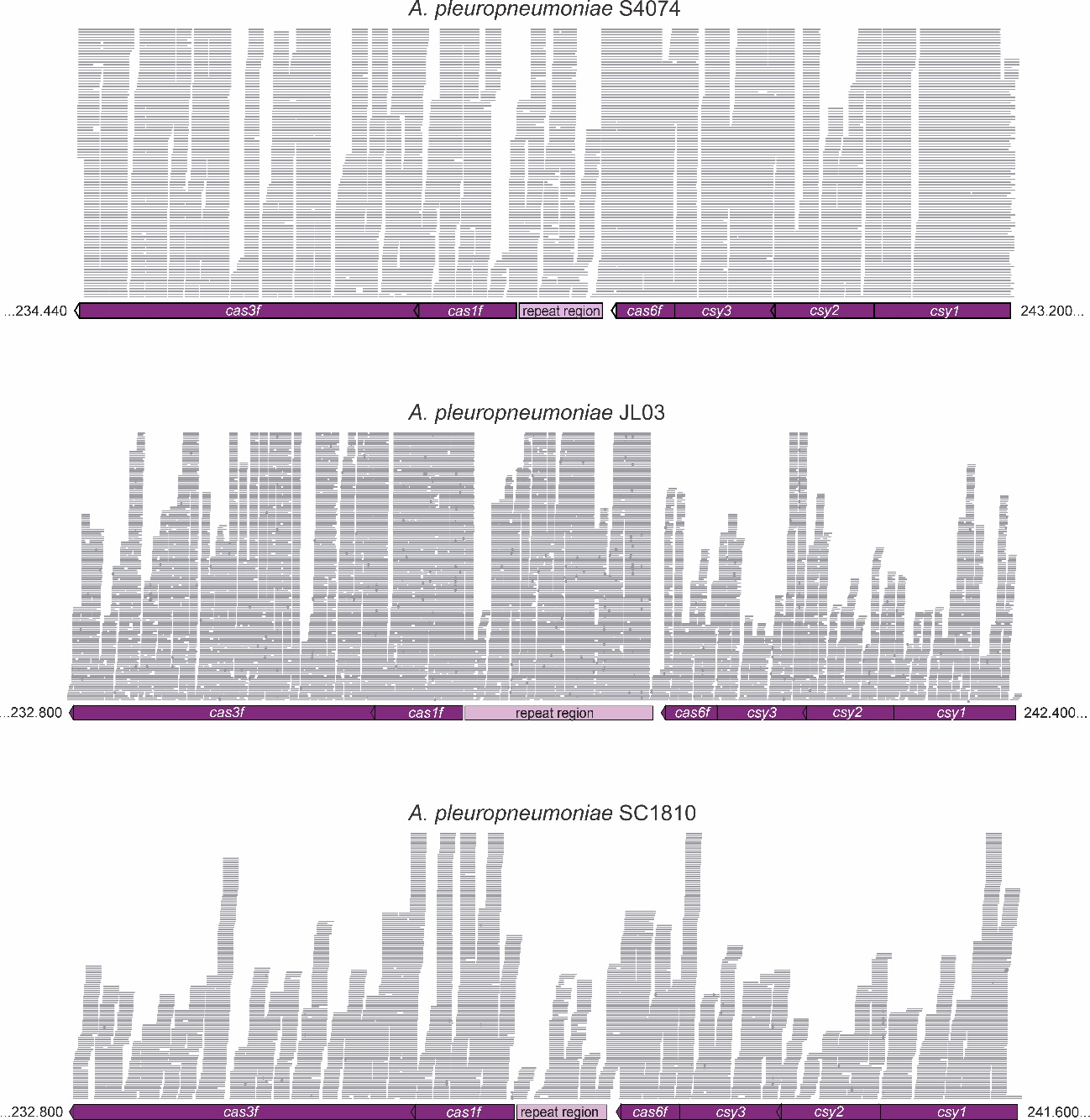


**Supplementary figure S2. Expression of CRISPR system in *A. pleuropneumoniae*.** RNA-Seq data from *A. pleuropneumoniae* strains S4074 (top), JL03 (middle), and SC1810 (bottom) display the expression of CRISPR loci, including spacers, across their genomes. Visualization was performed using Artemis.


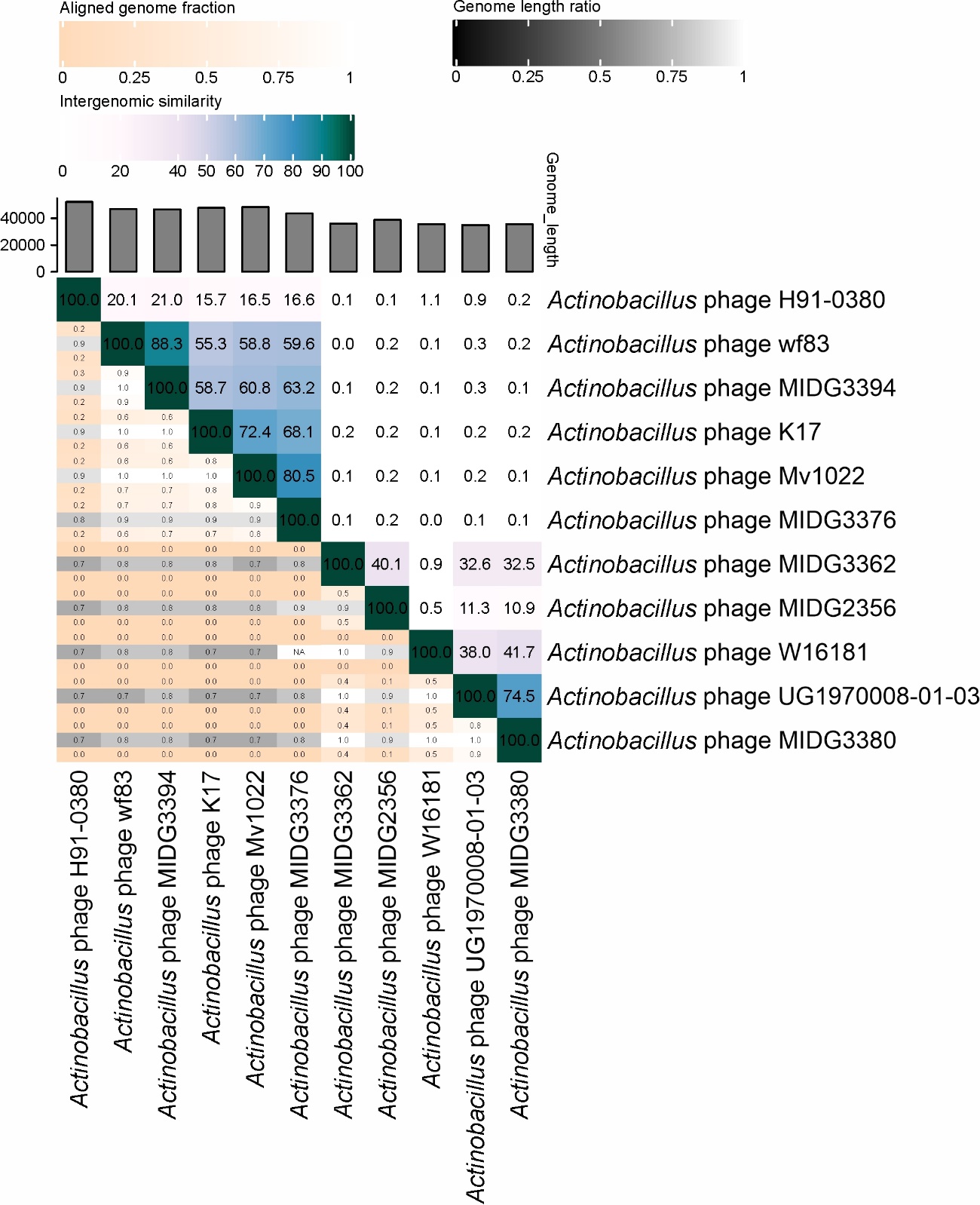


**Supplementary figure S3** - **Genome similarity map among the putative novel phages.** Heatmap generated by VIRIDIC displaying the percentage identity between pairs of newly identified putative phages. The values within the cells indicate genomic similarity, illustrating the proximity levels among the analyzed phage sequences.


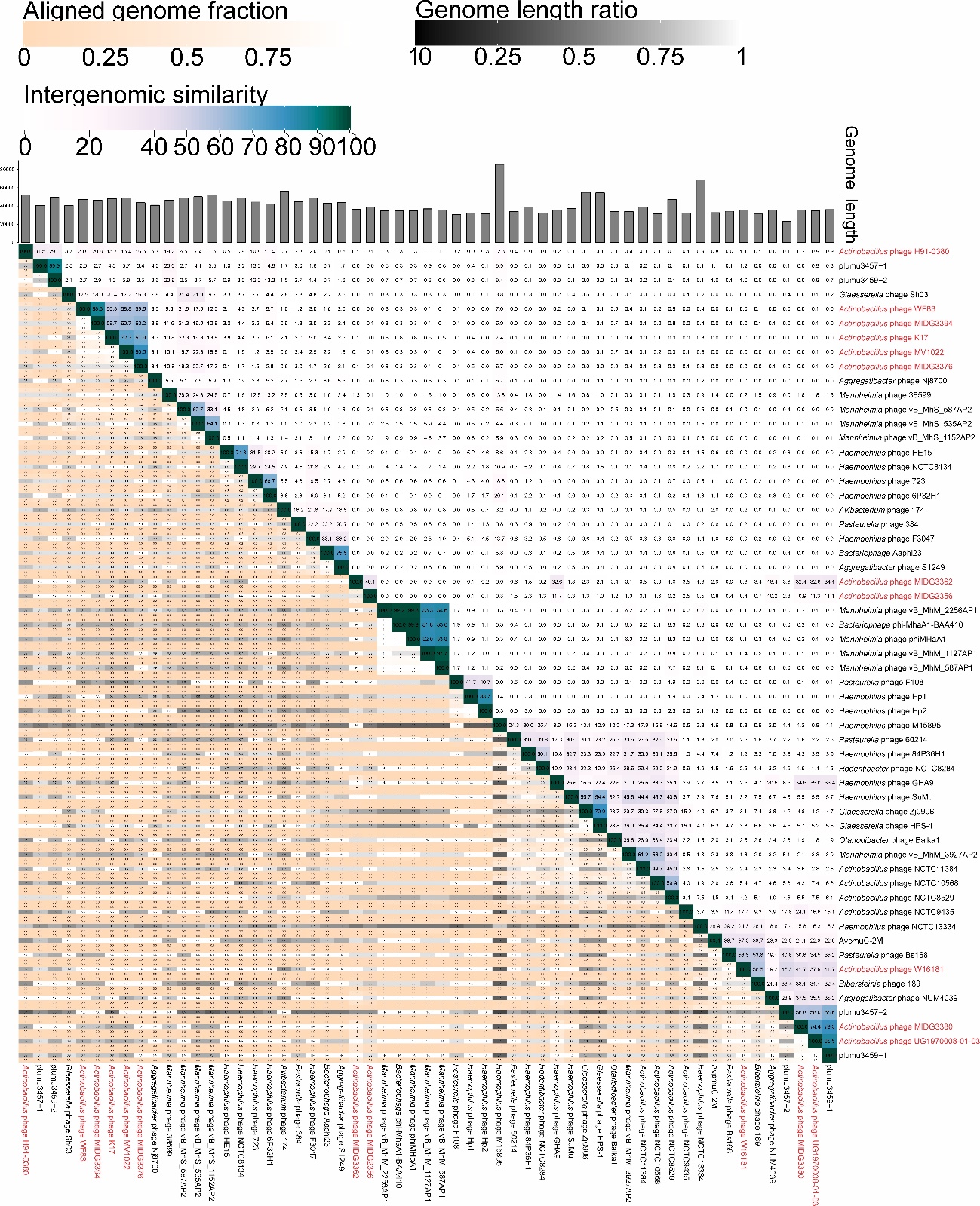


**Supplementary figure S4. Intergenomic similarity map of putative novel phages and previously described phages from Pasteurellaceae.** Heatmap generated by VIRIDIC displaying the percentage identity between pairs of newly identified putative phages (highlighted in red) and previously described phages. The values within the cells indicate genomic similarity, providing a comparative view of the proximity levels between the novel phages and known phages.


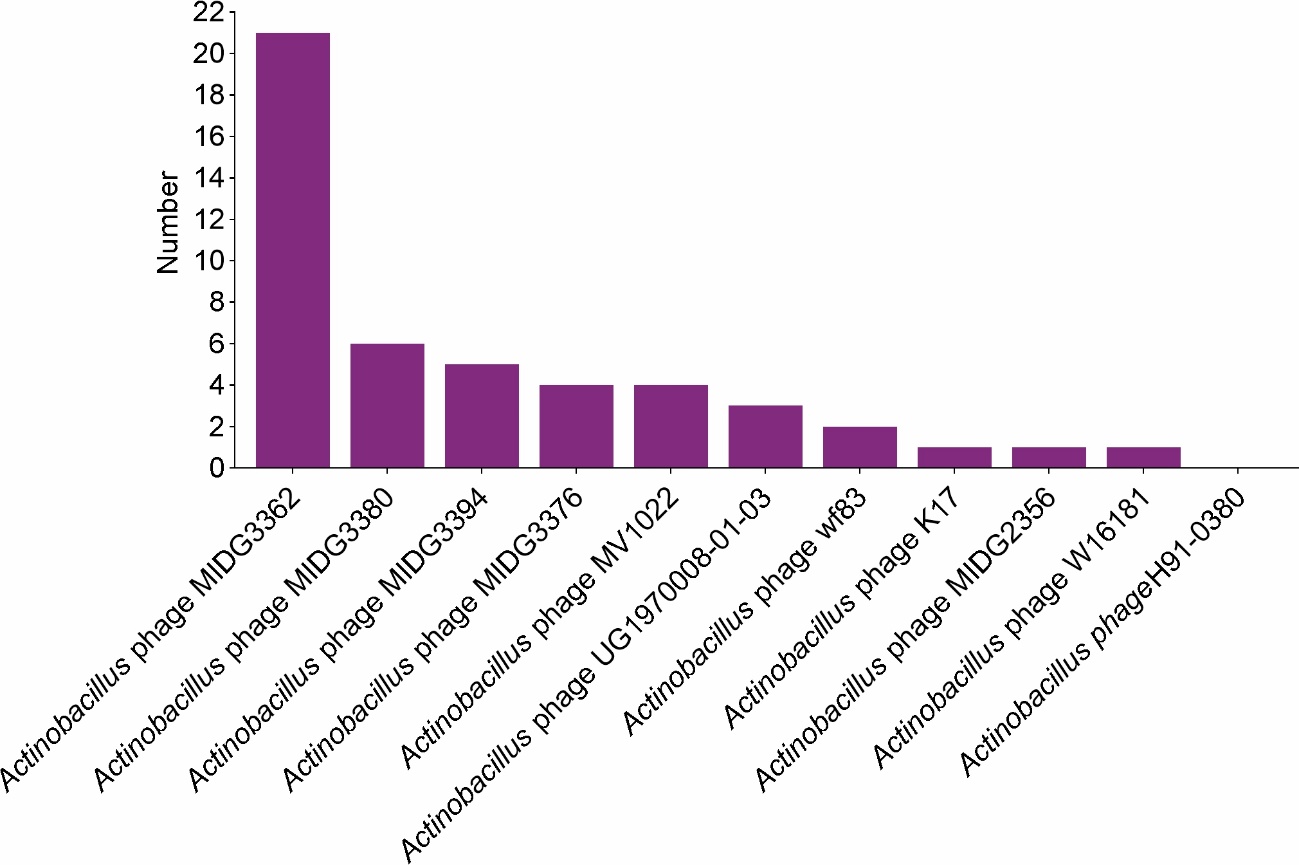


**Supplementary figure S5.** **Distribution of novel phages across *A. pleuropneumoniae* genomes.** Bar graph illustrating the presence of putative novel phages identified in this study across up to 23 different *A. pleuropneumoniae* genomes. This widespread occurrence underscores the potential significance of these phages in the genomic landscape of this species.
